# High Glucose Increases Metallothionein Expression in Renal Proximal Tubular Epithelial Cells

**DOI:** 10.1155/2011/534872

**Published:** 2011-09-22

**Authors:** Daisuke Ogawa, Masato Asanuma, Ikuko Miyazaki, Hiromi Tachibana, Jun Wada, Norio Sogawa, Takeshi Sugaya, Shinji Kitamura, Yohei Maeshima, Kenichi Shikata, Hirofumi Makino

**Affiliations:** ^1^Department of Medicine and Clinical Science, Okayama University Graduate School of Medicine, Dentistry and Pharmaceutical Sciences, Okayama 700-8558, Japan; ^2^Department of Diabetic Nephropathy, Okayama University Graduate School of Medicine, Dentistry and Pharmaceutical Sciences, Okayama 700-8558, Japan; ^3^Department of Brain Science, Okayama University Graduate School of Medicine, Dentistry and Pharmaceutical Sciences, Okayama 700-8558, Japan; ^4^Department of Dental Pharmacology, Okayama University Graduate School of Medicine, Dentistry and Pharmaceutical Sciences, Okayama 700-8558, Japan; ^5^CMIC Company, Ltd., Tokyo 113-0034, Japan; ^6^Center for Innovative Clinical Medicine, Okayama University Hospital, Okayama 700-8558, Japan

## Abstract

Metallothionein (MT) is an intracellular metal-binding, cysteine-rich protein, and is a potent antioxidant that protects cells and tissues from oxidative stress. Although the major isoforms MT-1 and -2 (MT-1/-2) are highly inducible in many tissues, the distribution and role of MT-1/-2 in diabetic nephropathy are poorly understood. In this study, diabetes was induced in adult male rats by streptozotocin, and renal tissues were stained with antibodies for MT-1/-2. MT-1/-2 expression was also evaluated in mProx24 cells, a mouse renal proximal tubular epithelial cell line, stimulated with high glucose medium and pretreated with the antioxidant vitamin E. MT-1/-2 expression was gradually and dramatically increased, mainly in the proximal tubular epithelial cells and to a lesser extent in the podocytes in diabetic rats, but was hardly observed in control rats. MT-1/-2 expression was also increased by high glucose stimulation in mProx24 cells. Because the induction of MT was suppressed by pretreatment with vitamin E, the expression of MT-1/-2 is induced, at least in part, by high glucose-induced oxidative stress. These observations suggest that MT-1/-2 is induced in renal proximal tubular epithelial cells as an antioxidant to protect the kidney from oxidative stress, and may offer a novel therapeutic target against diabetic nephropathy.

## 1. Introduction

Diabetic nephropathy is a leading cause of end-stage renal disease, and many mechanisms have been proposed to explain the pathogenesis of renal injury in diabetes [1]. Recent studies have shown that hyperglycemia may induce oxidative stress by increasing reactive oxygen species (ROS) generation in the diabetic kidney [2–4] and that overexpression of the antioxidant superoxide dismutase 1 attenuated diabetic nephropathy in streptozotocin (STZ)-induced and *db*/*db* diabetic mice [5, 6]. Therefore, ROS could be an important mediator of diabetic nephropathy, and protection from ROS might offer a valuable therapeutic strategy to treat diabetic nephropathy. 

Metallothionein (MT) is an intracellular metal-binding protein with a low-molecular mass (6-7 kDa) and a high cysteine content (20 of 61-62 amino acids). Its major isoforms, MT-1 and -2 (MT-1/-2), are widely distributed throughout the body [7, 8]. Since MT-1/-2 expression is significantly upregulated by overload of essential trace metals (e.g., Zn and Cu), it plays an important role in heavy metal detoxification and essential metal homeostasis [9, 10]. In addition, MTs have been shown to act as nonspecific free radical scavengers [11, 12], suggesting that they exert antioxidant activities in various diseases, including diabetic nephropathy.

We and other investigators have demonstrated that MTs have neuroprotective effects in mouse models of Parkinson's disease [13–15]. In contrast, the role of MTs in the pathogenesis of diabetic nephropathy is poorly understood. Several studies reported that renal expression of MT is increased in STZ-induced diabetic rats [16], diabetic BB rats [17], and *ob*/*ob* diabetic mice [18]. However, the distribution of MTs in the diabetic kidney and the mechanisms by which MTs are induced in diabetes are poorly understood. Therefore, in the present study, we investigated the expression and localization of MT-1/-2 during the development of diabetic nephropathy and explored the mechanism by which MT-1/-2 expression was induced by high glucose in the kidney.

## 2. Materials and Methods

### 2.1. Experimental Protocol

Male Sprague Dawley rats were purchased from Charles River (Yokohama, Japan). Five-week-old rats were divided into two groups: (1) nondiabetic control rats (control; *n* = 6) and (2) STZ-induced diabetic rats (DM; *n* = 6). Diabetes was induced by peritoneal injection of 200 mg/kg STZ (Sigma-Aldrich Corp., MO) in citrate buffer (pH 4.5). Blood glucose was measured by the glucose oxidase method at 3 days after STZ injection and only rats with blood glucose concentrations >16 mmol/L were used in the study. All rats had free access to standard diet and tap water. All procedures were performed according to the Guidelines for Animal Experiments at Okayama University Medical School, Japanese Government Animal Protection and Management Law (No. 105) and the Japanese Government Notification on Feeding and Safekeeping of Animals (No. 6). Rats were sacrificed at 1, 2, or 8 weeks after inducing diabetes. We measured body weight, hemoglobin A1c (HbA1c), and 24-h urinary albumin excretion (UAE) at 1, 2, and 8 weeks. The kidneys were removed, weighed, and fixed in 10% formalin for periodic acid—methenamine silver (PAM) staining, and parts of the remaining tissues were embedded in optimal cutting temperature compound (Sakura Finetechnical, Tokyo, Japan) and frozen immediately in acetone cooled on dry ice.

### 2.2. Immunofluorescent Staining of MT-1/-2 in Rat Kidney

Immunofluorescent staining was performed as previously described [19]. Renal expression of MT-1/-2 was detected using mouse anti-MT-1/-2 antibody (Dako, Carpinteria, CA) followed by Alexa Fluor 594 goat anti-mouse IgG (Invitrogen, Carlsbad, CA). To determine whether MT-1/-2 was localized in podocytes or proximal tubular epithelial cells, the sections were counterstained with guinea pig antinephrin antibody (Fitzgerald, Concord, MA) or rabbit antiaquaporin 1 antibody (Millipore, Billerica, MA), followed by Alexa Fluor 488 goat anti-guinea pig IgG or anti-rabbit IgG (Invitrogen), respectively. Fluorescence images were obtained using a fluorescence microscope (BX51; Olympus, Tokyo, Japan).

### 2.3. Cell Culture and Treatment

mProx24 cells, a murine renal proximal tubular epithelial cell line derived from C57BL/6J adult mouse kidney [20], were cultured in Dulbecco's modified Eagle's medium (Sigma-Aldrich Corp.) supplemented with 1000 mg/L D-glucose, 10% fetal bovine serum, 100 U/mL penicillin, and 100 mg/mL streptomycin at 37°C in 5% CO_2_. To evaluate the effect of high glucose on MT expression, the cells were serum-starved by culture in 0.5% FBS for 24 h, then stimulated with 4500 mg/L D-glucose (high glucose) or D-mannitol (Sigma-Aldrich Corp.) for 24 h. For antioxidant treatment, the cells were pretreated with vitamin E (Sigma-Aldrich Corp.) at concentration ranges from 20 to 200 nM for 24 h, then stimulated with high glucose for 24 h. Individual experiments were repeated at least three times with different lots or preparations of cells. 

### 2.4. Quantitative Analyses of MT-1 Gene and MT-1/-2 Protein Expression in mProx Cells

RNA was isolated from mProx cells using an RNeasy Mini kit (Qiagen, Valencia, CA). Single-strand cDNA was synthesized from the extracted RNA using a RT-PCR kit (Perkin Elmer, Foster City, CA). To evaluate the mRNA expression of MT-1 in mProx24 cells, quantitative RT-PCR (qRT-PCR) was performed using StepOnePlus (Applied Biosystems, Tokyo, Japan) and FastStart SYBR Premix Ex Taq II (Takara Bio Inc., Otsu, Japan). The primers for the MT-1 gene (upstream 5′-TCTAAGCGTCACCACGACTTCA-3′ and downstream 5′-GTGCACTTGCAGTTCTTGCAG-3′) were purchased from Takara Bio Inc. Each sample was analyzed in triplicate and normalized for GAPDH mRNA expression. Immunofluorescent staining of MT-1/-2 protein was performed as described above. The immunofluorescence intensity in cultured mProx cells was calculated using the formula, *x* (density) × positive area (*μ*m^2^), using Lumina Vision software (Mitani Corporation).

### 2.5. Statistical Analysis

All values are means ± SEM. Statistically significant differences between groups were examined using one-way ANOVA followed by Scheffé's test. Values of *P* < 0.05 were considered statistically significant.

## 3. Results

### 3.1. MT-1/-2 Expression Was Increased in Diabetic Kidney

**Figure 1:**
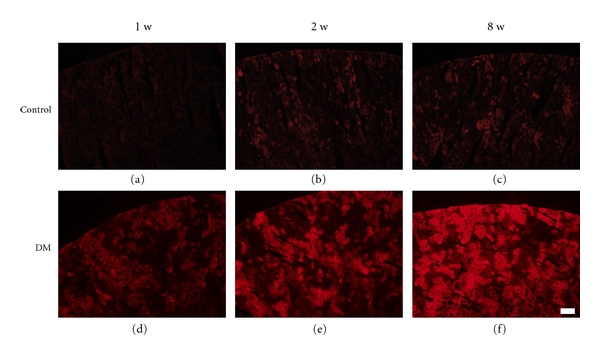
MT-1/-2 expression in the kidney. Diabetes was induced by injection of streptozotocin, and kidneys were obtained at 1 (a and d), 2 (b and e), or 8 (c and f) weeks after inducing diabetes. Immunofluorescent staining was performed as described in *Materials and Methods*. MT was strongly expressed in the renal cortex of diabetic rats (d, e, f) and hardly expressed in control rats (a, b, c). The expression of MT-1/-2 was greater at week 8 than at weeks 1 and 2 after diabetes induction. Scale bar: 100 *μ*m.

**Figure 2:**
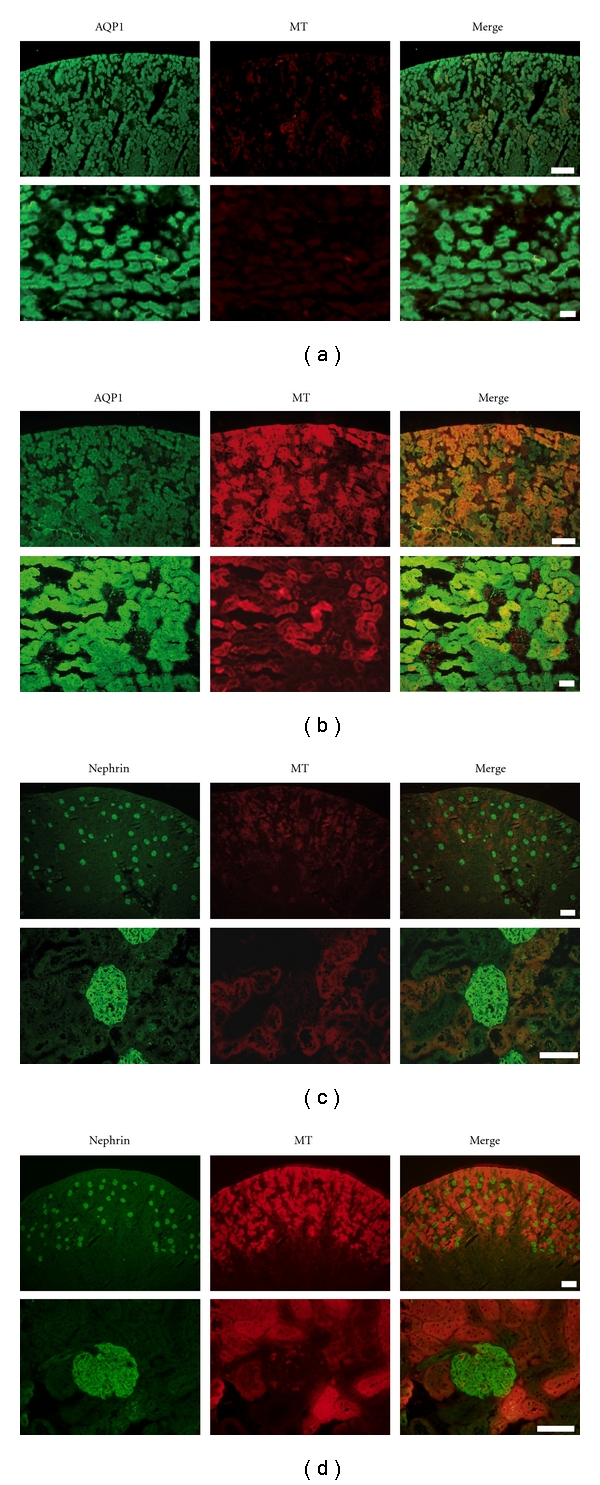
MT-1/-2 expression in podocytes and proximal tubular cells of the kidney. Immunofluorescent staining was performed as described in *Materials and Methods*. Eight weeks after inducing diabetes, MT-1/-2 was predominantly expressed in the proximal tubular epithelial cells of the kidney (b) and weakly expressed in podocytes (d) in the kidney of diabetic rats. In control rats, MT-1/-2 was weakly expressed in proximal tubular epithelial cells (a), but hardly in the podocytes (c). AQP1: aquaporin 1, MT: MT-1/-2. Scale bar: upper panels, 200 *μ*m; lower panels, 50 *μ*m.

MT-1/-2 expression was observed in the renal cortex from 1 week after the induction of diabetes. Its expression increased gradually and was strongly upregulated at week 8 (Figure 1,(d),(e),(f)). In contrast, MT-1/-2 was hardly detected in the kidney of control rats (Figure 1, (a),(b),(c)). Renal sections counterstained with antiaquaporin 1 and antinephrin antibodies revealed that MT-1/-2 expression was predominantly localized in the proximal tubular epithelial cells (Figure 2(b)), and to a lesser extent in the podocytes of the diabetic kidneys (Figure 2(d)). In control rats, MT-1/-2 was weakly expressed in the proximal tubular epithelial cells (Figure 2(a)), but not in the podocytes (Figure 2(c)). Body weight, kidney weight, UAE, and HbA1c are shown in Table 1. Diabetic rats had a significantly lower body weight and higher kidney weight per body weight at 8 weeks, but not at 1 and 2 weeks after the induction of diabetes. Similarly, The UAE and HbA1c level in the diabetic rats was significantly higher than in the control rats at 8 weeks, but not at 1 and 2 weeks. Glomerular hypertrophy and mesangial matrix expansion, but not interstitial changes and tubular atrophy were observed in the diabetic rats as compared with control rats at 8 weeks (data not shown). 

### 3.2. High Glucose Increased MT-1/-2 Expression in mProx24 Cells

**Figure 3:**
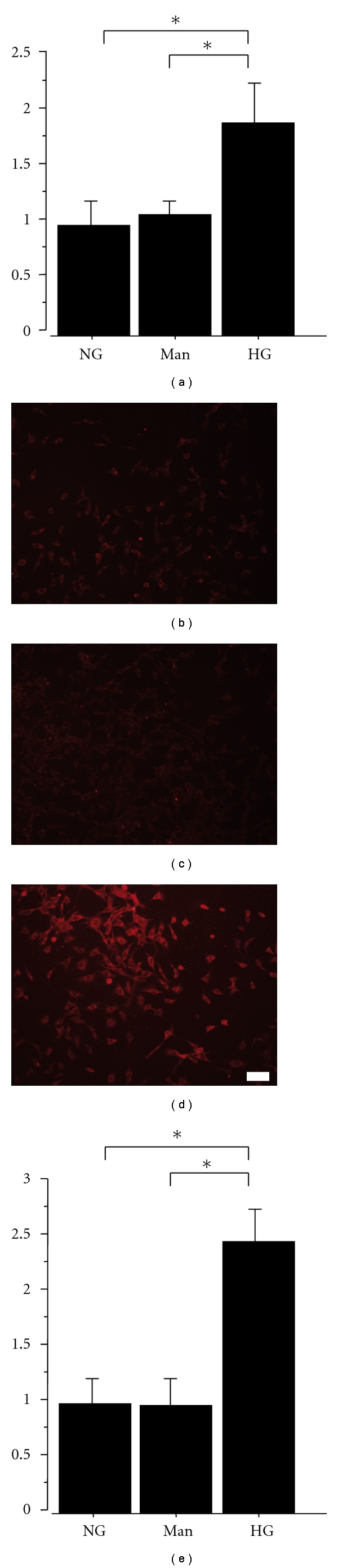
High glucose increases MT-1 mRNA and MT-1/-2 protein expression. mProx24 cells were serum-starved for 24 h before stimulation with high glucose or mannitol. (a) Cells were harvested after 24 h, and MT-1 mRNA expression was analyzed by qRT-PCR in three independent experiments and normalized for GAPDH. (b–e) MT-1/-2 protein expression was determined by immunofluorescent staining with anti-MT-1/-2 antibody 24 h after stimulation followed by densitometric analysis. Results are means ± SEM of three independent experiments. *P < 0.05 versus high glucose; NG: normal glucose; Man: mannitol; HG: high glucose. Scale bar: 100 *μ*m.

qRT-PCR analyses revealed that exposure to the high glucose medium significantly increased MT-1 mRNA expression in mProx24 cells compared with normal glucose medium (Figure 3(a)). Similarly, high glucose, but not mannitol, significantly increased MT-1/-2 protein expression in mProx24 cells (Figures 3(b)–3(e)). These data indicate that high glucose increases the mRNA and protein expression of MT-1/-2 in mProx24 cells.

### 3.3. MT-1/-2 Expression Was Suppressed by Vitamin E

**Figure 4:**
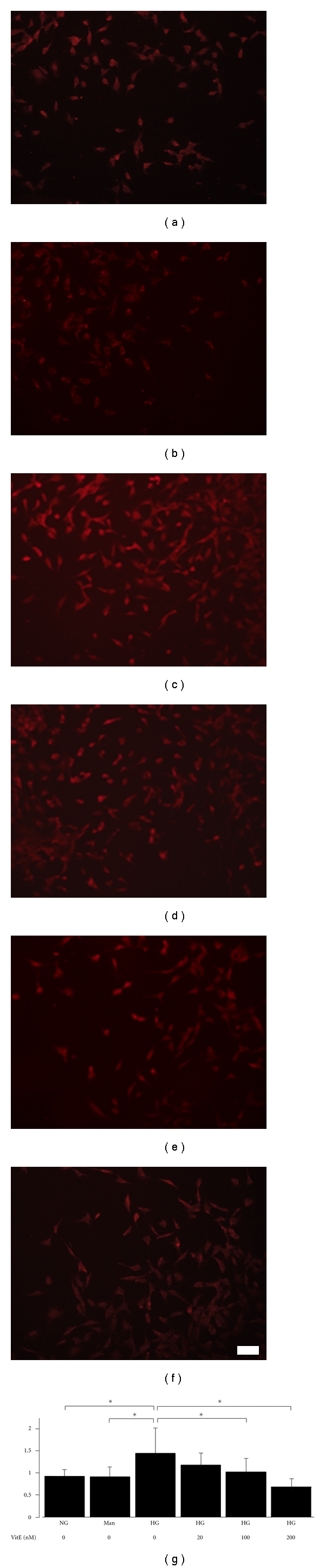
Vitamin E suppresses high glucose-induced MT-1/-2 expression. mProx24 cells were serum-starved and pretreated with vehicle or vitamin E for 24 h before stimulation with high glucose or mannitol. MT-1/-2 expression was determined by immunofluorescent staining. MT-1/-2 expression was not increased by mannitol (b) compared with normal glucose (a), but was increased by high glucose (c). High glucose-induced MT-1/-2 expression was attenuated by vitamin E pretreatment in a dose-dependent manner (d: 20 nM; E: 100 nM; F: 200 nM). The cells depicted are representative of three independent experiments. (g) Densitometric quantification of MT-1/-2 immunofluorescence. Results are means ± SEM of three independent experiments. *P < 0.05 versus high glucose; NG: normal glucose; Man: mannitol; HG: high glucose; Vit E: vitamin E. Scale bar: 100 *μ*m.

It is well known that high glucose increases the generation of ROS in various cells. To investigate the mechanism by which MT is induced by ROS in the high glucose condition, we examined the effects of an antioxidant, vitamin E, on MT-1/-2 expression in mProx24 cells. As shown in Figure 4, high-glucose-stimulated MT-1/-2 expression was significantly attenuated by vitamin E in a dose-dependent manner (Figure 4). Accordingly, these findings suggest that ROS generated by high glucose induces MT-1/-2 expression in the proximal tubular epithelial cells of the kidney.

## 4. Discussion

There is increasing evidence from experimental and clinical studies to suggest that oxidative stress plays a critical role in the pathogenesis and progression of diabetic complications [21]. Since MT is a potent, endogenous and inducible antioxidant in various tissues [11, 12], we hypothesized that MT may be induced and act as an antioxidant in STZ-induced diabetic kidneys. Here, we found that high glucose induces the expression of MT-1/-2 mainly in proximal tubular epithelial cells and, to a lesser extent, in podocytes in rat kidneys. MT-1/-2 was dramatically expressed in renal proximal tubular epithelial cells within 1 week after inducing diabetes and gradually increased to week 8. MT-1/-2 expression seems to correlate with glucose level, but not with UAE, HbA1c, interstitial abnormalities. To our knowledge, this is the first report describing the localization and expression of MT-1/-2 in the diabetic kidney. 

To elucidate the mechanism by which diabetes induces MT-1/-2 expression in proximal tubular epithelial cells, we investigated the effects of high glucose stimulation on mProx24, a murine renal proximal tubular epithelial cell line. We detected increased MT-1 mRNA and MT-1/-2 protein expression in the high glucose condition and found that high glucose-induced MT-1/-2 expression was suppressed by pretreatment with the antioxidant vitamin E. Vitamin E is well known to have high biological activity to protect cells from the propagation of free radical reactions [22, 23], thus we chose vitamin E in this study. These data suggest that ROS and oxidative stress, which are induced by high glucose, may be involved in the induction of MT-1/-2. Although several studies have shown that MT protein expression is increased in the kidney of diabetic animals [16–18], the cellular distribution of MTs has not been addressed. Our data provide the first evidence for the expression profile of MT-1/-2 in the diabetic kidney. We speculate that MT-1/-2 is highly induced in proximal tubular epithelial cells in compensation for oxidative stress induced by high glucose.

Our study has potential limitations. First, we speculated that MT-1/-2 expression was upregulated by ROS, but further studies are needed to elucidate the underlying mechanisms. Although Zn is known to induce the gene and protein expression of MTs [24], this essential trace element is unlikely to be involved in our findings because the same chow was provided to the control and diabetic rats. In this study, we showed that high-glucose-stimulated MT-1/-2 expression was attenuated by vitamin E *in vitro*, but we have no data about diabetic rats treated by vitamin E. MT-1/-2 expression in the diabetic state may differ between cells and tissues, and the mechanisms by which other antioxidants regulate the expression of MT remain unclear. Further studies are needed to elucidate these issues. Second, it is still controversial whether site-specific induction of MT plays an important role in diabetic nephropathy. Podocyte-specific overexpression of MT reduced diabetic nephropathy in transgenic mice [25]. However, no studies have investigated whether MT expression in proximal tubular epithelial cells has a protective effect in diabetic animal models. Therefore, diabetes models using MT-knockout mice are needed to answer this question.

In conclusion, renal ROS, which are induced by diabetes, upregulate MT-1/-2 expression in proximal tubular epithelial cells of the kidney. Our results suggest that MT-1/-2 might be a novel therapeutic target to treat diabetic nephropathy.

## Figures and Tables

**Table 1:** Metabolic data at 1, 2, and 8 weeks after inducing diabetes.

		1 week	2 week	8 week
Body weight (g)				
Control		204 ± 6.3	241 ± 10.4	380 ± 13.3
Diabetic		198 ± 4.7	225 ± 11.5	248 ± 16.6*
Kidney weight (mg/g BW)				
Control		5.8 ± 0.4	5.6 ± 0.8	4.5 ± 0.7
Diabetic		5.9 ± 0.6	6.1 ± 1.0	6.7 ± 0.9*
UAE (*μ*g/day)				
Control		110 ± 7.3	121 ± 8.1	137 ± 14.7
Diabetic		116 ± 5.7	125 ± 9.4	458 ± 24.5*
HbA1c (%)				
Control		3.7 ± 0.4	3.8 ± 0.6	3.8 ± 0.5
Diabetic		3.8 ± 0.3	4.3 ± 0.7	7.8 ± 0.9*

Data are means ± SEM; *P < 0.05 versus the control group. BW: body weight; UAE: urinary albumin excretion; HbA1c: hemoglobin A1c.
